# Autophagy Induced during Pancreatitis Promotes *KRAS*-Dependent Transformation in the Pancreas

**DOI:** 10.3389/fonc.2016.00226

**Published:** 2016-10-26

**Authors:** Juan L. Iovanna

**Affiliations:** ^1^Centre de Recherche en Cancérologie de Marseille (CRCM), INSERM U1068, CNRS UMR 7258, Aix-Marseille Université et Institut Paoli-Calmettes, Parc Scientifique et Technologique de Luminy, Marseille, France

**Keywords:** VMP1, pancreatic cancer, pancreatitis, autophagy, chloroquine, *KRAS*

## Abstract

Pancreatitis is an inflammatory disease that both facilitates and accelerates the transformation of pancreatic cells upon activation of the *KRAS* oncogene. Autophagy is proposed to be one of the cellular mechanisms contributing to pancreatic carcinogenesis, especially during initial stages in which the *KRAS* oncogene appears to play a key role. Autophagy is also strongly induced during pancreatitis by the overexpression of *VMP1*. We recently developed a genetically engineered mouse model in which the VMP1 protein is induced simultaneously with the activation of the oncogene *Kras^G12D^* specifically in the pancreas, by the addition of doxycycline to a water drink. Using this sophisticated animal model, we can affirm that pancreatic autophagy, induced during pancreatitis by the overexpression of *VMP1*, promotes the development of precancerous lesions when induced by the mutated *KRAS*. In addition, the treatment of these mice with chloroquine, an inhibitor of autophagic flux, reverses the effects of VMP1 in pancreatic cancer induced by the *KRAS* oncogene. Overall, these results bear both mechanistic and biomedical relevance for further understanding and potentially targeting pathways that are critical for initiating pancreatic carcinogenesis, particularly if associated with pancreatitis.

## Pancreatic Ductal Adenocarcinoma

Pancreatic ductal adenocarcinoma (PDAC) is the fourth leading cause of cancer death in the Western world, with prediction curves demonstrating it will become the second leading cause of death by cancer in 2030, just after lung cancer (1). Both the initiation and progression of this pathology result from the interaction of complex genetic events with multiple less characterized factors (2, 3). Genetic alterations that contribute to the pathogenesis of pancreatic adenocarcinoma have been widely studied and definitively determined. Among these alterations, oncogenic mutations in the *KRAS* gene have been frequently detected (more than 90% of cases), not only in the established disease but also in preneoplastic lesions known as pancreatic intraductal neoplasia (PanINs). Activation of the oncogene *KRAS* signals pancreatic cells to undergo acinar-to-ductal metaplasia, an essential step in the formation of premalignant lesions, which together with the inactivation of tumor suppressor genes, such as *CDKN2A, TP53*, and *SMAD4*, allow the progression of premalignant lesions to invasive cancer (4). As the activating mutation in the *KRAS* oncogene is almost systematically associated with PDAC, its role in cancer development has been the subject of numerous studies (5).

Autophagy has been proposed as a cellular process contributing to pancreatic carcinogenesis, particularly in the initial stages in which the *KRAS* oncogene is a key element (6–9). Indeed, activation of the pathway controlled by the *KRAS* oncogene generates a metabolic stress, characterized by a temporary deficit in energy, which must be compensated by an increase in metabolism, through activation of autophagy (6–10). Although this concept appears clear and simple, the role of autophagy in protumor or antitumor development is still debated in the context of PDAC, since multiple factors appear to modulate this process, such as regulatory pathways, the genomic status of transformed pancreatic cells, as well as the physiological and pathological contexts in which the process is enabled (11, 12).

## Pancreatitis-Associated Autophagy Promotes the Protumoral Effect of the *KRAS* Oncogene

Pancreatitis, an inflammatory disease of the pancreas, enables and accelerates the transformation of pancreatic cells when the *KRAS* oncogene is activated (13). Exactly how pancreatitis promotes the development of PDAC is a fundamental question in the field of pancreatology, which has not yet been clearly answered. However, this has been partly answered by studies showing that the systematic activation of autophagy during pancreatitis, often for the protection of pancreatic cells, decreases disease progression and aids the recovery phase (14, 15). We have demonstrated that induction of autophagy in pancreatic acinar cells is accompanied by the overexpression of the *VMP1* gene. VMP1 mRNA encodes a transmembrane protein that we cloned in 2002 due to its extraordinary pancreatic activation during the acute phase of pancreatitis (16). Overexpression of VMP1 triggers autophagy in numerous types of cells (16–19). Concerning its mechanistic activity, VMP1 is involved in the formation of the phagophore (18) following a direct interaction with beclin 1 (17), TP53INP2, a scaffold protein (20), and possibly its homolog, TP53INP1 (21). The main physiological role of autophagy during pancreatitis is the removal of damaged organelles to maintain cellular homeostasis and ensure improved survival of pancreatic cells (22). It is likely that the protective effect of autophagy during the acute phase of the disease is at least partly related to the sequestration of zymogen granules that contain digestive enzymes responsible for autodigestion during pancreatitis. This may have a dual effect on pancreatic cells: first, zymophagy (autophagy of zymogen granules) could reduce the availability of digestive enzymes, which when released into the pancreatic parenchyma destroys the pancreatic gland by necrosis; second, these organelles could meet the unique metabolic needs that accompany cell growth during the regeneration phase (23).

## Autophagy Induced by Overexpression of VMP1 Enhances Transformation of Pancreatic Cells

**Figure 1:**
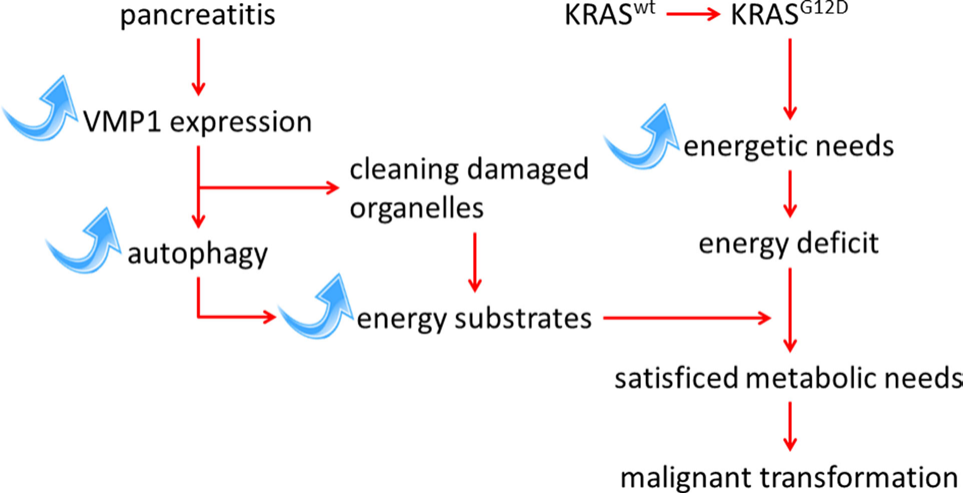
**Schematic representation of the interaction between *KRAS*-mediated transformation in PDAC and autophagy induced by the pancreatitis-associated protein VMP1 pathways**.

It is interesting to note that the expression of VMP1 is also transcriptionally activated by the mutated *KRAS* oncogene through a mechanism dependent on GLI3 and p300 (24). The *KRAS* oncogene possibly induces VMP1 expression to meet the increased energy needs of the cell during the transformation process. Expression of the VMP1 protein, and its triggered autophagy, is therefore induced and maintained by mutation of the *KRAS* oncogene, which is strongly reinforced during the course of pancreatitis. The most likely hypothesis is that autophagy induced by pancreatitis, and mediated by overexpression of VMP1, provides the energy required of cells harboring an activating mutation in the *KRAS* oncogene, therefore allowing their transformation. To test this hypothesis, we have recently developed an animal model wherein the genetically modified VMP1 protein is induced simultaneously with the activation of the oncogene *Kras^G12D^* specifically in the pancreas, by the addition of doxycycline to a water drink (25). This model was developed with the objective to first assess the effects of overexpressed VMP1 on initiation of pancreatic cancer, and second, to define the role of pharmacological inhibitors of autophagy in the development of pancreatic cancer. The results of these experiments in mice affirm our hypothesis that autophagy, induced by overexpressing VMP1 in the pancreas, significantly increases the protumor effect of the *KRAS* oncogene (Figure 1). In addition, we demonstrated that chloroquine, a classical inhibitor of autophagic flux (26), can reverse the effect of VMP1 overexpression on pancreatic cancer induced by the *KRAS* oncogene in a preclinical trial using our mouse model (25). Overall, these observations support the idea that pathways activated by pancreatitis, particularly those regulating autophagy, can promote pancreatic carcinogenesis. Finally, the results support the concept that inhibition of autophagy could be used to prevent the progression of pancreatic pre-tumoral lesions to pancreatic cancer.

## Mechanisms of Action of VMP1

In light of these clinically relevant results, it is important to review and discuss the identified functions of the VMP1 protein, which will consequently improve the interpretation of its role in pancreatic tumor progression. For example, it has been established that this protein is involved in the initiation of autophagy since cells engineered to be deficient in VMP1 have high levels of PtdIns3P and trigger autophagic signaling by the resulting aberrant endoplasmic reticulum, with subsequent recruitment of ATG18 and other autophagic proteins (19). In addition, although ULK1 and ATG5 are separated in the genetic hierarchy during autophagy, both proteins accumulate synchronously within punctate structures containing VMP1, followed by recruitment of ATG14, ZFYVE1, and WIPI1 (27). Moreover, VMP1 protein directly binds to the BH3 motif of beclin 1 to induce the formation of a complex with hVps34, a key phosphatidylinositol-3 kinase class III regulator of autophagy, on the site where autophagosomes are generated. Importantly, the interaction between beclin 1 and VMP1 proteins leads to the dissociation of the Bcl-2 protein with beclin 1, therefore increasing intracellular levels of beclin 1 available to induce autophagy (28). In addition, the presence of the VMP1 protein regulates the formation of autophagosomes by shortening the training time of the omegasome and therefore significantly accelerating autophagic flux (18). Finally, the production of cells inactivated for VMP1 protein in *Dictyostelium* revealed a massive accumulation of protein aggregates, both poly- and multi-ubiquitinated, containing the autophagic markers ATG8 counterparts and p62 but presenting strong defects in autophagy process. Altogether, these observations demonstrate that expression of the stress protein VMP1 is essential for unloading cells of these protein aggregates by autophagy (29) and recycling them to provide the energy substrate required by the cell under these stress conditions.

It is also important to discuss the broader role that autophagy plays in the development of PDAC as it is so complex and varied. Indeed, it was previously demonstrated that autophagy participates in the transition from mitosis to senescence (30), and certain molecules can induce both autophagy and senescence, such as kinase ULK3 (30). Senescence is known to be an important anticancer pathway set up in response to the oncogenic activation of mutated *KRAS*. In this context, senescence enhanced by activation of autophagy might partially inhibit the oncogenic effect of the *KRAS* oncogene rather than increase it. Furthermore, activation of autophagy in certain tissues, either dependent or independent of VMP1 overexpression, can act as an antiapoptotic factor, according to the biological circumstances (31, 32). Finally, as mentioned earlier, the oncogenic activation of *KRAS* induces a strong metabolic stress to cells due to their exceptional energy requirements that can be partially counterbalanced with the contribution of energy sources through the activation of autophagy. Autophagy can therefore play important roles in either promoting or, on the contrary, antagonizing the development of PDAC, depending on the activated intracellular pathways by cells harboring *KRAS* mutations. This possibly explains the contrasting results reported in the literature on the role of autophagy in cancer. Another important note is that a large majority of these studies were performed *in vitro*, therefore the cellular environment has not, or only partially, been taken into account, possibly causing a bias in data interpretation. Regarding the pancreatic autophagy induced by VMP1 overexpression in mice, we have established that the development of pancreatic precancerous lesions is associated with a significant reduction of apoptosis with a concomitant increase in cell proliferation (25). In other words, autophagy is clearly a pro-tumor cellular event, at least in this context.

Importantly, autophagy has been considered an important mediator of the resistance to radiotherapy and chemotherapy, at least with particular anticancer drugs and for certain cancers (33, 34), although this point still remains controversial. Nevertheless, the fact that cancer treatments systematically induce autophagy has now been clearly established (35). However, the mechanism by which autophagy is involved in resistance to cancer treatments seem to be initiated by the removal of damaged intracellular organelles to improve cell viability. Furthermore, autophagy has also been reported as a mediator of cell death induced by chemotherapy in several cancers (36). Although the mechanism by which autophagy induces cell death is not yet clearly established, it appears to be mediated by the activation of caspase 3 (36). Therefore, in line with such knowledge, co-treatment with chloroquine appears to enhance the effect of many anticancer drugs *in vitro* as well as in some preclinical models (37–41), although a clinical study has yet to confirm its benefit as a co-treatment.

## Conclusion and Perspectives

In conclusion, many aspects concerning the role of autophagy during PDAC development are still not clearly defined. However, we can confirm that pancreatic autophagy induced during pancreatitis through the overexpression of VMP1, a protein associated with pancreatitis, promotes PanINs when activated by the *KRAS* oncogene. In addition, inhibition of autophagic flux by chloroquine almost completely abolishes the *KRAS* pro-tumor effect in the pancreas. Overall, these results bear both mechanistic and biomedical relevance for further understanding and potentially targeting those pathways critical for initiating pancreatic carcinogenesis, particularly if associated with pancreatitis. In the near future, it will be necessary to take into account not only the role of autophagy activation in transformed cells but also in the stromal non-transformed cells. Recently, it was clearly evidenced that the activation of autophagy in cancer-activated fibroblast (CAF cells) is an essential mechanism to produce and secrete non-essential amino acids into the microenvironment, which serves as a major source of energy for transformed cells (42). This may be the starting point of a novel time in which the autophagy may be considered as the fuel source for other cells. All in all, these facts are revealing a more complex scenario than suspected and therefore are opening news ways for treating diseases in which autophagy seems to be strongly involved, such as PDAC. An interesting observation to be noted was recently pointed out by Guo and colleagues who demonstrated that the loss of VMP1 expression in colorectal cancer is associated with a poor prognosis and aggressiveness of the cancer cells. In addition, *in vitro* assays revealed that colon cancer-derived cells in which VMP1 was knocked down gained significant aggressive properties in regards to proliferation and invasion. Remarkably, *in vivo* studies revealed a higher number of formed nodules in mice after intraperitoneal injection of VMP1 knocked down cells (43). Another recent work reports that approximately 10% of esophageal adenocarcinomas present a RPS6KB1–VMP1 gene fusion as a recurrent event. Notably, esophageal adenocarcinoma cases harboring RPS6KB1–VMP1 fusions exhibited significantly poorer overall survival as compared to fusion-negative cases. Mechanistically, the RPS6KB1–VMP1 fusion protein promotes cell growth *in vitro*, but it is ineffective in triggering autophagy (44). Altogether, these studies suggest that the role of VMP1, and perhaps autophagy, in cancer development and progression is organ or context dependent.

## Author Contributions

The author confirms being the sole contributor of this work and approved it for publication.

## Conflict of Interest Statement

The author declares that the research was conducted in the absence of any commercial or financial relationships that could be construed as a potential conflict of interest.
